# Osteopontin promotes a cancer stem cell-like phenotype in hepatocellular carcinoma cells via an integrin–NF-κB–HIF-1α pathway

**DOI:** 10.18632/oncotarget.3113

**Published:** 2015-02-25

**Authors:** Lei Cao, Xiaoyu Fan, Wei Jing, Yingchao Liang, Rui Chen, Yingying Liu, Minhui Zhu, Rongjie Jia, Hao Wang, Xueguang Zhang, Yanyun Zhang, Xuyu Zhou, Jian Zhao, Yajun Guo

**Affiliations:** ^1^ School of Medicine, Shanghai Jiao Tong University, Shanghai, 200025, People's Republic of China; ^2^ International Joint Cancer Institute, The Second Military Medical University, Shanghai, 200433, People's Republic of China; ^3^ Changhai Hospital, The Second Military Medical University, Shanghai, 200433, People's Republic of China; ^4^ PLA General Hospital Cancer Center, PLA Postgraduate School of Medicine, Beijing, 100853, People's Republic of China; ^5^ Clinical Immunology Laboratory, The First Affiliated Hospital of Suzhou University, Suzhou, 215007, Jiangsu, People's Republic of China; ^6^ Beijing Key Laboratory of Cell Engineering & Antibody, Beijing, 100853, People's Republic of China

**Keywords:** Cancer stem cell, hepatocellular carcinoma, osteopontin, stemness

## Abstract

There is increasing evidence to suggest that hepatocellular carcinomas (HCCs) are sustained by a distinct subpopulation of self-renewing cells known as cancer stem cells. However, the precise signals required for maintenance of stemness-like properties of these cells are yet to be elucidated. Here, we demonstrated that the level of oncoprotein osteopontin (OPN) in tumor cells of the edge of bulk tumors was significantly correlated with the clinical prognosis of patients with HCC. OPN was highly expressed in side population fractions of HCC cell lines, as well as in dormant cells, spheroids and chemo-resistant cancer cells, all of which are considered as having stemness-like cellular features. Depletion of OPN in HCC cell lines resulted in a reduction in the proportion of side population fractions, formation of hepato-spheroids, expression of stem-cell-associated genes and decreased tumorigenecity in immunodeficient mice. Mechanistically, OPN was demonstrated to bind to integrin α_v_β_3_ and activate the transcription factor NF-κB, which resulted in upregulation of *HIF-1α* transcription and its downstream gene, *BMI1*, to mediate maintenance of the stemness-like phenotype. Suppression of the α_v_β_3_–NF-κB–HIF-1α pathway decreased OPN-mediated self-renewal capabilities. Levels of OPN protein expression were significantly correlated with HIF-1α protein levels in HCC tumor tissue samples. OPN might promote a cancer stem cell-like phenotype via the α_v_β_3_–NF-κB–HIF-1α pathway. Our findings offer strong support for OPN requirement in maintaining stem-like properties in HCC cells.

## INTRODUCTION

Hepatocellular carcinoma (HCC) is the fifth most prevalent cancer worldwide and as such presents a major global health burden. The current definitive treatment for HCC is surgical resection combined with chemotherapy [1, 2]. Despite major advances in the development of treatment approaches for the disease, improvements in disease-free survival and long-term prognosis for patients with HCC remain unsatisfactory [3, 4].

Cancer stem cells (CSCs) are a subset of tumor cells with the capability to self-renew and differentiate into a diverse range of cell types that contribute to tumor growth and recurrence, as well as conferring resistance to chemotherapeutic agents [5]. CSCs were first identified in leukemias and have since been found in range of solid tumors, including breast, brain, prostate, melanoma and colon cancers [5]. In the hematopoietic compartment, a side population of stem cells that express the receptor tyrosine kinase, Tie2, are quiescent and antiapoptotic [6]. Similar to this side population of hematopoietic stem cells (HSCs), CSCs are also quiescent, which may account for these cells being able to evade the effects of chemotherapy and radiation treatments [7, 8].

In the case of HCC, research directed towards understanding the role of CSCs has attracted a large amount of attention. A number of studies that have characterized CSCs in HCC have focused on cells that express surface markers, such as CD133, CD44, CD90, EpCAM, CD24 and CD13 [8–13]. Moreover, a side population of cells that are antipoptotic, express ‘stemness genes’ and exhibit pluripotent potential have been identified in HCC tissues and might function as CSCs [14].

The function of oncogenes in CSCs and the pathways that regulate self-renewal and tumorigenesis in HCC are unclear; and development of targeted therapies aimed at oncogenes or pathways that are pathogenic in both primary and metastatic HCC continues to pose a challenge. The oncoprotein osteopontin (OPN) is a chemokine-like, extracellular matrix protein that binds to integrins and to members of the CD44 receptor family on which is expressed on multiple HCC cell types [15–17]. Upon binding, various kinases, including PI3K, and MAP3K14, and several transcription factors such as nuclear factor-kappa B (NF-kB), AP-1 and Ets-1 are activated leading to OPN-induced tumor growth, angiogenesis, tumor metastasis and inhibition of apoptosis [18–21].

Previously, we have demonstrated that OPN functions in HCC to promote tumor growth and metastasis through induction of mitochondria-mediated apoptosis [17]. OPN is expressed in the hematopoietic stem cell compartment where it is involved in negative regulation of HSC proliferation [22, 23]. In glioma, OPN and CD44 share a similar perivascular expression profile, and OPN was found to promote development of glioma stem cell-like phenotypes through its action as a CD44 ligand [24]. However, little is known regarding the function of OPN in CSCs in the context of HCC.

Here, we sought to explore the function of OPN in HCC-associated CSCs and to determine the mechanism by which OPN might contribute to tumor progression and survival in HCC. Our findings suggest that overexpression of OPN promotes development of cells with stemness-like characteristics in HCC, via modulation of the integrin αvβ3–NF-kB–hypoxia-inducible factor-1 alpha (HIF-1α) signaling axis. These data identify a novel role for OPN as an effector that mediates development of progenitor cells in the liver and of CSCs that drive progression of HCC.

### OPN expression is correlated with side-population fraction

A side-population fraction of cells with a high self-renewal and tumorigenic properties has been identified in several cancer types [6, 14, 25]. Although some controversy exists regarding the function of these cells, one hypothesis is that cells in the side-population could be CSCs [26]. Supporting this hypothesis, cells that were isolated from side-populations of HCC cell lines demonstrated CSC-like properties, such as increased chemo-resistance, and were associated with enhanced tumorgenicity *in vi*vo [27].

**Figure 1:**
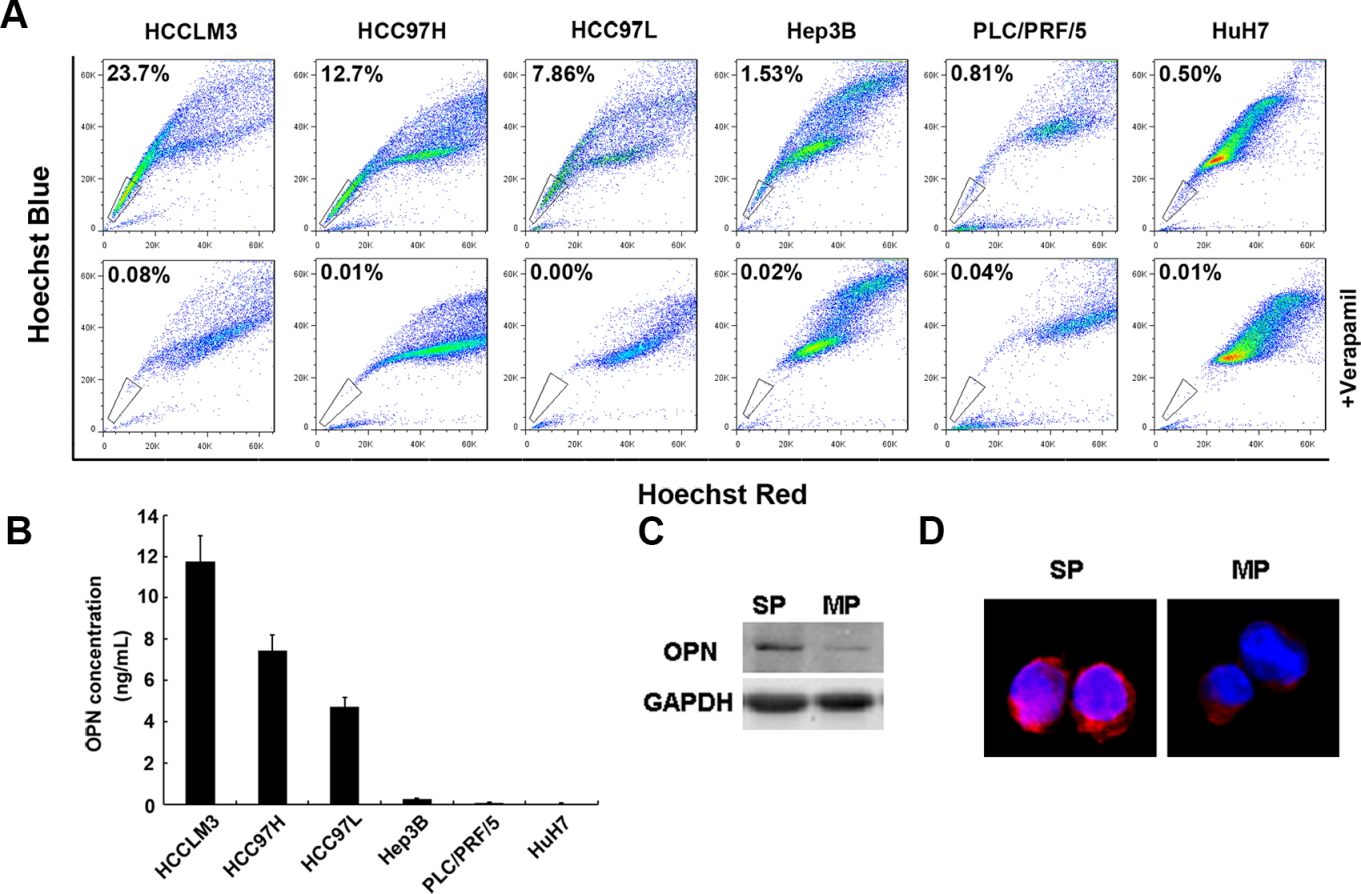
OPN expression is high in a side population of HCC cells **(A)** The side population fraction of HCC cells is higher in metastatic HCC cell lines (HCCLM3, HCCC97H, HCC97L) than in non-metastatic HCC cell lines (Hep3B, PLC/PRF/5, HuH7). Side population cells are indicated by the gate. **(B)** Secreted OPN protein is increased in metastatic HCC cell lines compared with non-metastatic HCC cell lines. **(C)** Cells in the side population fractions have higher levels of OPN expression than cells in the main population fraction either by western blot assay or **(D)** by immunofluoresence. Magnification ×40.

Side-population fractions of six HCC cell lines, including HCCLM3, HCC97H, HCC97L, Hep3B, PLC/PRF/5 and HuH7 were analyzed (Figure 1A). In metastatic HCC lines (HCCLM3, HCC97H and HCC97L), the side-population fractions ranged from 23.7% to 7.86%. By comparison, proportion of side-population cells in non-metastatic HCC lines (Hep3B, PLC/PRF/5 and HuH7) were markedly lower and ranged from 1.53% to 0.5%. We then assessed the relationship between side-population fractions and levels of secreted OPN in HCC cell lines. Consistent with a role for OPN in driving metastasis, levels of secreted OPN were much higher in metastatic cell lines with high side-population fractions than those in the non-metastatic cell lines with low side-population fractions (Figure 1B). The self-renewal capacity of cells in the side and main populations of HCCLM3 cells was also examined. In both sphere-forming and colony-forming assays, cells in the side population fraction formed great numbers of both spheres and colonies than cells in the main population. This finding indicates that cells in the side population have greater self-renewal capacity than those in the main population of the HCCLM3 cell line (Supplementary Figures S1A and S1B). To further delineate the association of OPN production and cells in the side-population fraction, we sorted the side-population and main-population from HCCLM3 cells and assessed levels of OPN protein expression. Western blot revealed a great increase of OPN protein levels in side-population cells compared with main-population cells (Figure 1C). This finding was supported by semi-quantitative immunofluorescence analysis, which suggested that more OPN protein was present in side-population cells versus main-population cells (Figure 1D).

### OPN expression in tumor cells of the edge of bulk tumors is associated with increased tumor aggression and decreased survival in patients with HCC

**Figure 2:**
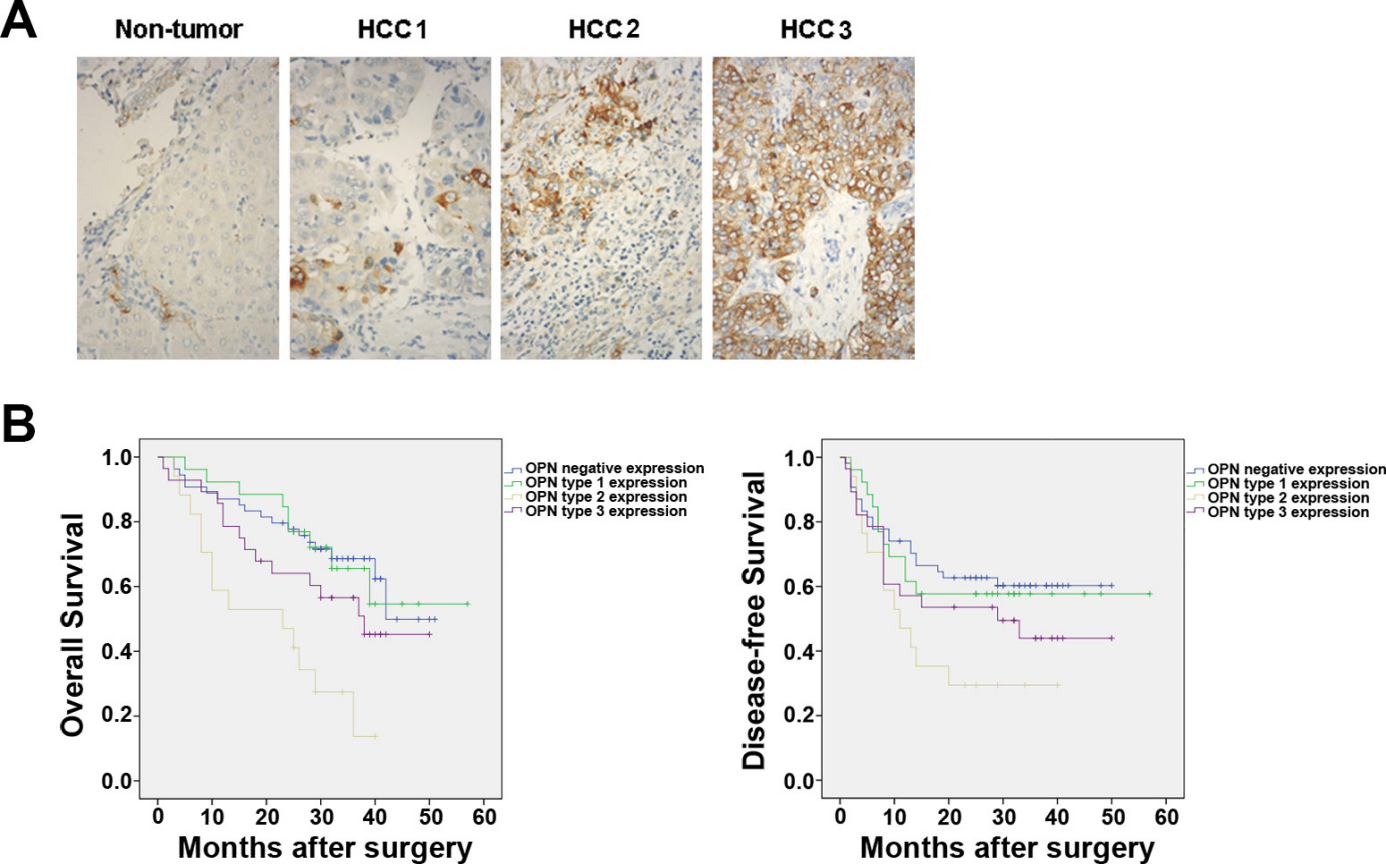
OPN expression is heterogeneous across HCC tumors **(A)** Representative samples of the three major expression patterns of OPN expression in human HCC tumor tissues samples stained for are shown. Type 1 (< 10% of cells are OPN-positive), type 2 (OPN localized to cells at the edge of the bulk tumor) and type 3 (constitutive expression of OPN in all tumor cells). Magnification ×40. **(B)** The disease-free and overall survival rates of subtypes of 125 patients with HCC compared with patients with tumors that showed OPN-negative expression or the indicated types of OPN expression. The numbers of cases of each type of OPN expression are 54 patients (OPN-negative), 26 patients (type-1-OPN expression), 17 patients (type-2-OPN-expression) and 28 patients (type-3-OPN expression).

We examined the expression of OPN protein in tissue samples resected from patients with HCC. In non-tumor tissues OPN was weakly expressed in cholangiocytes but in no other cells types, however, by contrast, OPN was highly expressed in bulk tumor tissues (Figure 2A). Furthermore, three distinct patterns of OPN expression were observed in HCC tumor sections: type 1 revealed OPN expression in less than 10% tumor cells and was distributed across the bulk of the tumor; type 2 expression was characterized by localization to tumor cells at the edge of bulk tumor cells, which is the region that invades the capsule; the type 3 pattern was marked by constitutive OPN expression in almost all tumor cells. These patterns revealed that OPN expression is heterogeneous across human HCC tumor specimens.

We then sought to determine the association, if any, between the classifications of OPN expression and the clinical presentation of patients with HCC (Supplementary Table S1). No significant associations were found for any of the patterns of OPN expression with gender, hepatitis B surface antigen (HbsAg), serum AFP level and cirrhosis (Table 1). However, only the type 2 OPN expression pattern was found to be significantly associated with the stage of HCC when compared to OPN-negative tumors (*P* = 0.022). Notably, patients with type 2 OPN expression experienced significantly higher recurrence rates following surgical resection than patients with negative OPN expression (*P* = 0.010).

**Table 1:** Relationship between OPN expression and clinicopathologic features of HCC patients

Feature	OPN expression				*P*		
	negative (*n* = 54)	type1 (*n* = 26)	type2 (*n* = 17)	type3 (*n* = 28)	type1 v.s. negative	type2 v.s. negative	type3 v.s. negative
Gender					0.281	0.48	0.677
Male	48	25	14	24			
Female	6	1	3	4			
Age(year)					0.025	0.872	0.951
<50	37	11	12	19			
≥50	17	15	5	9			
HbsAg					0.975	0.697	0.208
Positive	52	25	16	25			
Negative	2	1	1	3			
AFP(ng/ml)					0.764	0.746	0.523
≤400	23	12	8	14			
>400	31	14	9	14			
Cirrhosis					0.109	0.345	0.777
−	12	2	2	7			
+	42	24	15	21			
Tumor volume(cm3)					0.917	0.185	0.815
<5	16	8	8	9			
≥5	38	18	9	19			
AJCC stage					0.652	0.022	0.246
I–II	36	16	6	15			
III–IV	18	10	11	13			
recurrence					0.538	0.01	0.109
−	35	15	5	13			
+	19	11	12	15			
Death					0.598	0.01	0.854
−	32	17	4	16			
+	22	9	13	12			

Kaplan-Meier survival analysis revealed that patients with type 2 OPN expression patterns had significantly lower rates of disease-free survival and overall survival when compared to patients with tumors that were negative for OPN expression (*P* = 0.010 and *P* = 0.010, respectively; Figure 2B and Table 1). Together, these results suggest the expression of OPN in tumor cells of the edge of bulk tumors is associated with increased tumor aggression and decreased survival in patients with HCC.

### OPN is highly expressed in HCC cells with stem-like properties

**Figure 3:**
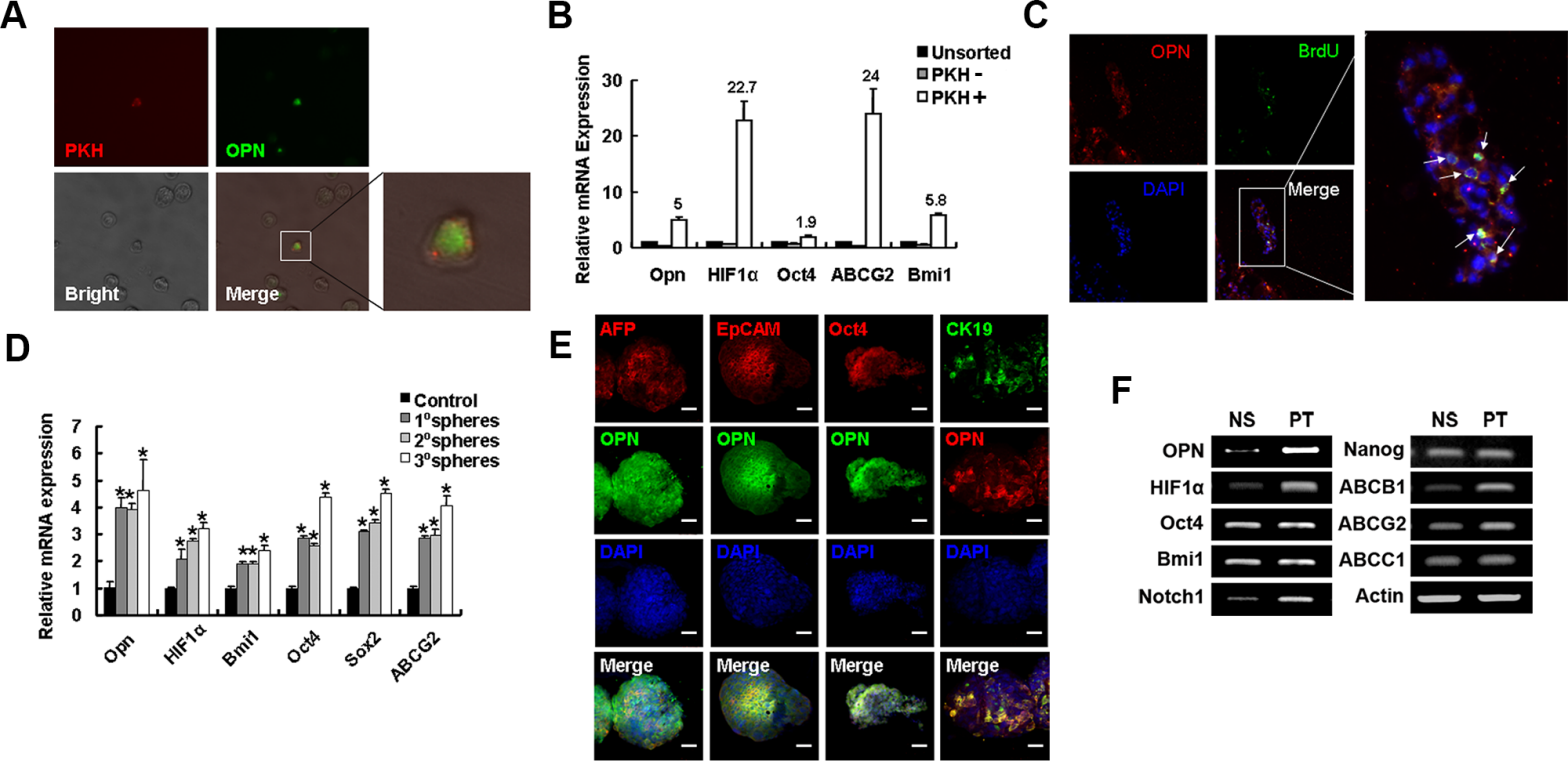
OPN is highly expressed in self-renewal cells **(A)** OPN staining of PKH labeled cells ex vivo 6 weeks after inoculating. Magnification ×20. The boxed area is magnified (×80) on the right to better visualize the PKH^+^ cell. **(B)** qPCR analysis was performed for OPN, HIF-1α, OCT4, ABCG2 and BMI1 in PKH^+^ and PKH^−^ and untreated cells. **(C)** OPN expression is high in cells that retained BrdU staining six weeks after tumor inoculation of nude mice. OPN (red), BrdU (green) and DAPI (blue). Original magnification ×10. Arrowheads indicate cells that coexpress OPN and BrdU. Boxed area ×40. **(D)** The expression of OPN, as well as stemness-related genes, including SOX2, OCT4, ABCG2, and BMI1, was increased with serial sphere formation assays compared to control adherent cells. **(E)** Representative confocal images of OPN costained with stem-cell-associated proteins AFP (red), EpCAM (red), OCT4 (red) or CK19 (green) and DAPI (blue) in Hep3B spheres. Scale bars represent 50 μm. **(F)** RT-PCR analysis of stemness-related genes and genes associated with chemo-resistance were compared in normal saline treated tumors and PT treated tumors.

Given that side-population HCC cells have striking similarities to stem cells and are a relatively dormant, we investigated whether OPN was also highly expressed in this population. Cells that retained the PKH26 label, which is indicative of the cells being dormant, in both *in vivo* and *in vitro* experiments demonstrated high expression of OPN (Figures 3A; Supplementary Figure S2). Furthermore, dormant cells demonstrated significantly higher expression of stem-cell-associated genes, including *OCT4*, *BMI1*, *HIF-1α* and the gene that encodes the drug resistant transporter ABCG2, compared to cells that did not retain the PHK26 label (Figure 3B). Strikingly, expression of *OPN* from PKH high positive cells was five-fold increase compared to that of cells from unsorted fractions and 13.9-fold greater than cells in the PKH negative fractions (Figure 3B). Further analysis of expression of OPN in quiescent cells was conducted using HCCLM3 cells that were xeno-transplanted into nude mice and the developing tumors labeled with BrdU. After six weeks of tumor growth, BrdU-label-retaining cells were typically observed at the edge of tumor foci and these cells were also found co-localized with staining for OPN (Figure 3C).

Spheres contain stem/progenitor cells, and the number of spheres formed upon serial passage under defined culture conditions is considered a reflection of the self-renewal capacity of stem/progenitor cells [28]. In HCCLM3 cells, accompanied with the up-regulation of several stem-cell-associated genes, including *OCT4*, *SOX2*, *BMI1*, *HIF-1α* and *ABCG2*, the expression of *OPN* was increased in serial spheres compared to adherent cells by a factor of approximately 4–4.5-fold (Figure 3D). Similar to the reported levels of mRNA transcripts, levels of OPN protein were also increased in spheres compared to adherent cells (Supplementary Figure S3A). Furthermore, Hep3B spheres were co-stained of OPN with other markers associated with hepatic stem cells or stem cells. The majority of cells in the Hep3B spheres expressed high levels of OPN protein, which were co-localized with several known hepatic stem cells markers, such as AFP, EpCAM and CK19, and the stem-cell-associated marker OCT4 (Figure 3E).

Given the hypothesis that CSCs are resistant to chemotherapeutic agents, we sought to investigate the expression of OPN in chemo-resistant HCC cells. In cultured HCC97L or Hep3B cells OPN protein levels were up-regulated following 72 hrs of exposure to either cisplatin (PT) or 5-fluorouracil (5-FU) (Supplementary Figure S3B). A xenograft model was established to generate and enrich for CSCs with increased chemo-resistance. The chemo-resistant tumors expressed high levels of stemness-associated genes, including *OCT4*, *BMI1* and *NOTCH1*, as well as chemo-resistant-related genes, such as *HIF-1α* (Figure 3F). Importantly, *OPN* mRNA levels were increased in the chemo-resistant tumors (Figure 3F). We also examined the expression of OPN in tumors by IHC and confirmed that OPN expression in chemo-resistant tumors was significantly up-regulated relative to control tumors (Supplementary Figure S3C).

Together, these data demonstrate that OPN is highly expressed in HCC cells with stem-like properties.

### OPN is critical to maintaining the CSC-like phenotype

**Figure 4:**
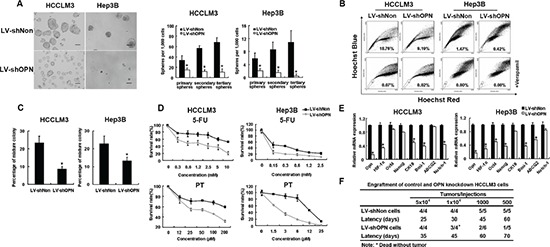
OPN knockdown reduced stem and progenitor characteristics of tumor cells **(A)** LV-shOPN cells generated a decreased number of series passaged spheroids compared with LV-shNon cells. Representative phase-contrast image of HCC spheroids derived from HCCLM3 and Hep3B cells are shown. Scale bars represent 100 μm. **(B)** Down-regulation of OPN decreased the proportions of side population cells in cultured HCCLM3 and Hep3B cells. **(C)** OPN knockdown reduced the percentage of mixed colonies in both HCCLM3 and Hep3B cell lines. **(D)** Knockdown of OPN in HCCLM3 and Hep3B resulted in reduced cellular survival 48 hrs after treatment with chemotherapeutic agents 5-FU and PT. **(E)** qRT-PCR analysis was performed for OPN, HIF-1α, OCT4, NANOG, CK19, BMI1, ABCG2 and NOTCH1 genes in LV-shNon versus LV-shOPN cells of HCCLM3 and Hep3B. Significant increases in expression of stemness-associated genes were observed in LV-shOPN groups. **(F)** Engraftment of control and OPN knockdown HCCLM3 cells. Experiments were performed in triplicate, and data were shown as mean ± SD. *P < 0.05.

Given the association of OPN expression with various CSC properties, such as localization to a side-population fraction, dormancy, sphere formation and chemo-resistance, we asked if depletion of OPN could influence the CSC-like characteristics of HCC cells. The knockdown efficiency of two shRNA constructs was assessed in HCCLM3 and Hep3B cell lines and the construct that target site 2 was chosen for further study (Supplementary Figures S4A). The effect of knocking-down OPN expression on self-renewal was assessed by anchorage-independent colony formation and sphere formation assays. HCC cells in which OPN expression was knocked-down exhibited a dramatically decreased ability to form colonies (Figure 4A and Supplementary Figures S4B and S4C). OPN knocked-down also resulted in generation of fewer and smaller spheres in each serial passage compared to control groups (Figure 4A). Analysis of side populations in OPN depleted HCCLM3 and Hep3B cells demonstrated reductions of 52% and 70% in proportions, respectively (Figure 4B).

HCC cells with self-renewal potential can differentiate to cells with different lineage-specific markers. We, therefore, assayed lineage markers at the single cell level in a mixture colony assay. Three types of colonies arose from the HCCLM3 cells: mixture colony, consisting of AFP^+^CK19^+^ and AFP^+^CK19^−^ cells; and two homogenous types of colonies, consisting of either AFP^+^CK19^−^ or AFP^−^CK19^−^ cells. Unlike the homogenous colonies, the mixed phenotype colony demonstrated that cells that form this type of colony are stem/progenitor cells with potential for bi-directional differentiation. Unlike the HCCLM3 cells, four types colonies arose from the Hep3B cells: mixed colony that consisted of the four cell types AFP^+^CK19^+^, AFP^+^CK19^−^, AFP^−^CK19^+^ and AFP^−^CK19^−^ cells; and each of the other three homogenous colonies, consisting of either AFP^+^CK19^−^, AFP^−^CK19^+^ or AFP^−^CK19^−^ cells. In the HCCLM3 control cells, the proportion of mixed colonies formed that represent a self-renewal ability was 24%, which was much higher than the 7.5% of the OPN-knockdown cells (Figure 4C). Likely, in Hep3B, the mixture colony containing all phenotypes was 23.4% in control group versus 12.7% in OPN knockdown group (Figure 4C). Representative images of the mixed colonies are shown in Supplementary Figure S4D. The finding that OPN-depleting diminished ability of HCC cells to form stem/progenitor cells suggests that OPN is a critical determinant in CSC-development in HCC.

We also investigated the effects of OPN-depletion on chemo-resistance in HCC cells. Compared with control cells, OPN-knockdown cells displayed significantly higher sensitivities to the two tested chemotherapeutic agents (*P* < 0.01; Figure 4D). In addition, we compared the expression of stemness-associated genes between OPN-knockdown cells and controls. The results showed that OPN-knockdown resulted in downregulation of *HIF-1α*, *OCT4*, *NANOG*, *CK19*, *BMI1*, and *NOTCH1* (Figure 4E).

Investigation of the role of OPN in tumorigenesis demonstrated that while control cells readily formed tumors within five weeks, the same number of OPN-knockdown cells failed to form palpable tumors within 11 weeks (Supplementary Figure S4E). Consistent with these findings, when cells were implanted into the flanks of SCID mice, control cells produced large tumors in 100% of mice; whereas only 25% mice that were injected with OPN-knockdown cells developed tumors at 12 weeks after transplantation (Figure 4F). Thus down-regulation of OPN attenuated the self-renewal capabilities of HCC cells *in vitro*, as well as limiting expression of stemness-associated genes and *in vivo* tumor formation.

### Secreted HCC-derived OPN maintains cancer cell stemness

It has been reported that plasma levels of OPN is significantly elevated in patients with HCC compared to healthy control individuals. Furthermore, the concentration of serum OPN is markedly increased with larger tumor burdens in patients with cancer [29]. We hypothesized that secreted OPN might have an important role in maintaining the stemness-like characteristics of HCC cells.

**Figure 5:**
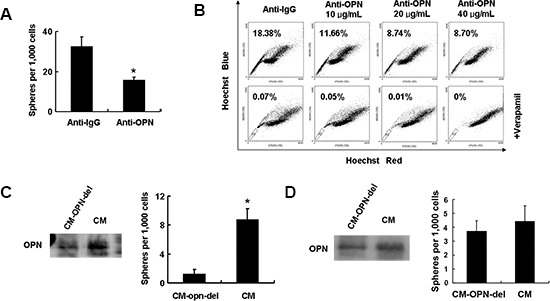
Secreted OPN from HCC cells maintains CSC-like phenotypes **(A)** OPN blockade leads to a reduction in spheroid formation in HCCLM3 cells. **(B)** The side population fraction of cells is reduced in a dose-dependent fashion with addition of increasing concentrations of neutralizing OPN antibodies to cultured HCCLM3 cells. **(C)** Secreted levels of OPN protein in OPN-deleted conditional media and control media from HCCLM3 culture supernatant. HL7702 cells cultured in OPN-deleted conditional media have decreased self-renewal ability in in vitro a sphere forming assay when compared to cells cultured in control conditional media. **(D)** No decrease in spheroid formation is apparent in OPN-depleted cells cultured in THP-1 conditioned media.

The effects of blocking OPN signaling on the self-renewal capacity of HCC cells were investigated. Addition of anti-OPN antibodies to HCCLM3 cells inhibited sphere formation (Figure 5A). Supplementation of different concentrations (10 μg/mL, 20 μg/mL and 40 μg/mL) of anti-OPN antibody to the media of cultured HCCLM3 cells resulted in reductions of the proportions of side population fractions from 18.8% to 8.72% (Figure 5B).

The effects of secreted OPN on self-renewal were further investigated by depleting extracellular OPN from conditioned media by immunoprecipitation. As shown in Figure 5C, OPN expression in OPN-depleted HCCLM3 conditioned media was substantially decreased compared with complete HCCLM3 conditioned media. The HL7702 cell line, which is an immortal human liver cell line, readily formed spheres when cultured with control HCCLM3 conditioned media. However, fewer spheres were observed in when HL7702 cells were cultured in the presence of OPN-depleted conditioned media (Figure 5C).

Since inflammatory cells in the tumor microenvironment also express OPN, we assessed the effects of secreted OPN derived from inflammatory cells on self-renewal. First, we activated THP-1 (a human monocytic cell line) cells by treatment with PMA in serum-free culture media and collected the conditioned media after 24 hrs. Extracellular OPN was depleted from the conditioned media as described above. Spheres formation of HL7702 cells in conditioned media was unaffected by the depletion of OPN, which suggested that OPN derived from inflammatory cells has limited impact on tumor stemness (Figure 5D).

### OPN supports cellular self-renewal via HIF-1α and BMI1

**Figure 6:**
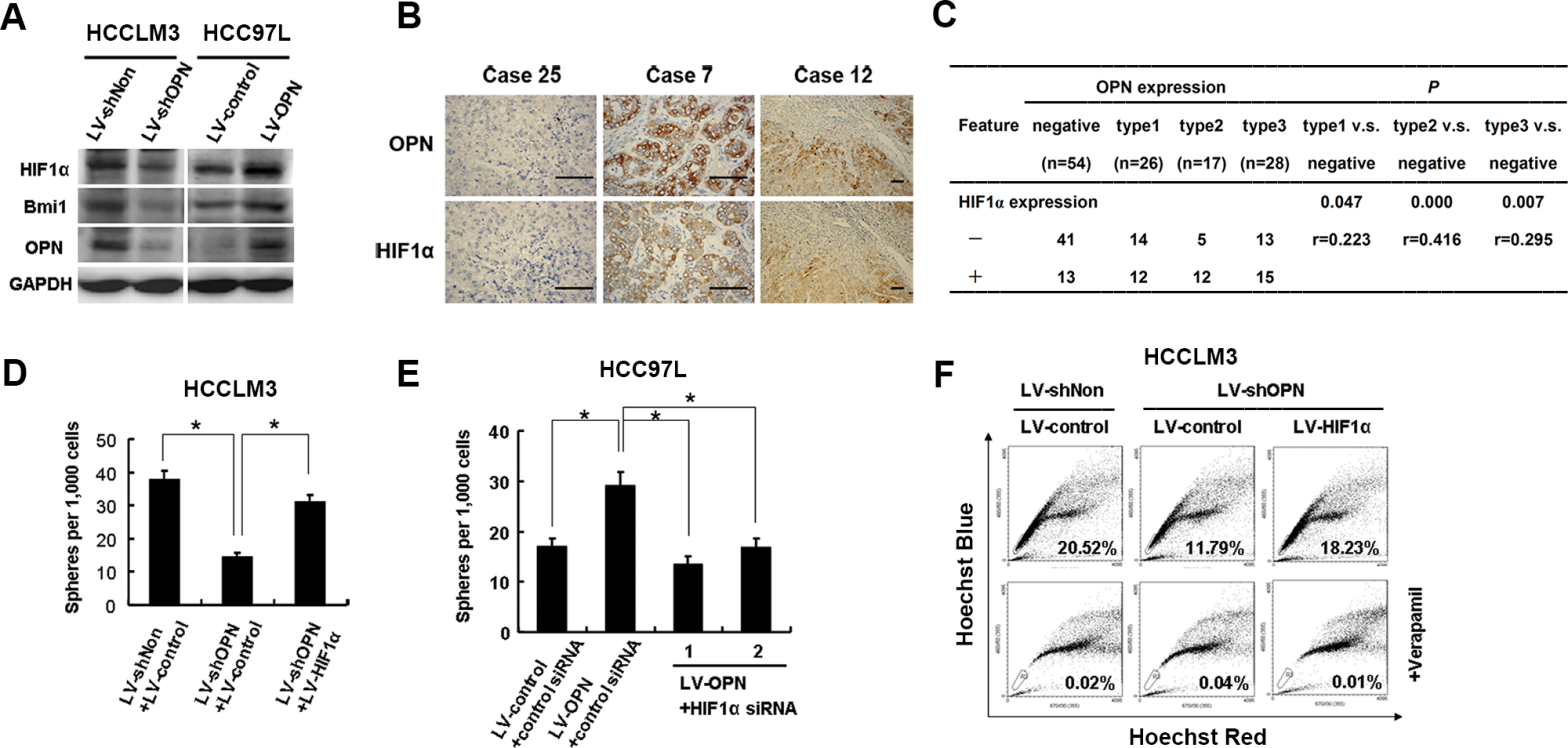
OPN signaling is mediated through HIF-1α-BMI1 **(A)** The effects of depletion (HCCML3 cells) and overexpression (HCC97L cells) of OPN on HIF-1α and BMI1 expression **(B)** OPN and HIF-1α expression is colocalized in HCC tumor tissues. Representative images of OPN and HIF-1α IHC staining in HCC tumor tissue sections are shown. Scale bar: 100 μm. **(C)** Correlation of OPN and HIF-1α expression in 125 HCC tumor tissue samples. Correlation analysis: Spearman's correlation. **(D)** Quantification of the sphere formation from HCCLM3 LV-shNon or LV-shOPN cells that are infected with LV-HIF-1α or control lentivirus. **(E)** Quantification of the spheroid formation from HCC97L cells that overexpress OPN treated and siRNAs targeting HIF-1α The data are reported as mean ± SD. *P < 0.05. **(F)** Quantification of side population fractions from HCCLM3 LV-shNon or LV-shOPN infected with LV-HIF-1α or control lentivirus.

To determine the major downstream mediators of OPN signaling in tumor initiation and self-renewal, we assessed the expression of stemness-associated genes in both HCCLM3 and Hep3B cell lines expressing OPN-targeting siRNA-LV constructs. Expression of both *HIF1α* and *BMI1* was consistently downregulated or upregulated in response to loss of OPN expression or overexpression of OPN, respectively (Figures 4E and 6A). We then examined the correlation between OPN and HIF-1α expression by IHC in tumor tissue samples from patients with HCC. Representative images of negative (case 25), type 2 (case 12) and type 3 (case 7) OPN expression patterns with corresponding HIF-1α expression are shown (Figure 6B). In the OPN-negative group, HIF-1α co-expression was found in 13 of 54 (24.1%) patient samples. However, in patients with type 2 and 3 OPN expression patterns, HIF-1α coexpression was found in 12 of 17 (70.6%) and 15 of 28 (53.6%) patient samples, respectively (Figure 6C). The correlation between OPN and HIF-1α expression was significant for patients with type 2 expression versus OPN-negative patients (*P* = 0.000, r = 0.416) and for patients with type 3 expression patterns versus OPN-negative patients (*P* = 0.007, r = 0.295) (Figure 6C).

To determine if OPN maintains tumor stemness and self-renewal characteristics by activating HIF-1α gene expression, we overexpressed HIF-1α in OPN-depleted HCCLM3 cells. HIF-1α overexpression resulted in an increase in the number of spheres formed and in the side population fraction, which was reduced in the absence of OPN expression (Figures 6D and 6F and Supplementary Figure S5A). Similarly, HIF-1α expression rescued the cell survival phenotype in response to treatment with PT or 5-FU (Supplementary Figure S5C).

An HIF-1α siRNA knockdown strategy was implemented to further elucidate the role of this protein in maintaining stemness characteristics. BMI1 expression was significantly down-regulated when HIF-1α expression was silenced, which is consistent with a report that BMI1 is activated under hypoxic conditions or by HIF-1α overexpression [30] (Supplementary Figure S5B). Sphere formation in cells deficient for HIF-1α was also decreased significantly compared to controls in HCC97L cells that overexpressed OPN (Figure 6E). Since BMI1 overexpression was observed in tumor-initiating cells and levels of *BMI1* mRNA were downregulated in response to OPN knockdown, we asked if BMI1 functioned downstream of OPN to mediate stemness in HCC cells. Overexpression of BMI1 in OPN-deficient cells reversed the negative effects of OPN depletion on sphere formation (Supplementary Figures S6A and S6B). Consistent with these findings, knock down of BMI1 protein levels resulted in a reduction of sphere formation in HCC97L cells that overexpressed OPN (Supplementary Figures S6C and S6D). These data indicate that HIF-1α and BMI1 function downstream of OPN to mediate stemness of HCC cells *in vitro*.

### The integrin α_v_β_3_–NF-κB–HIF1α axis mediates OPN-induced stemness

We sought to determine the transcription factor that regulates *HIF-1α* expression in the context of OPN signaling. NF-kB is a transcription factor that has previously been reported to bind to the HIF-1α promoter under conditions of reactive oxygen species stimulation [31]. Given that NF-kB is activated upon OPN-mediated apoptosis, we were interested to determine if NF-kB also mediates OPN-induced stemness in HCC cells [17].

**Figure 7:**
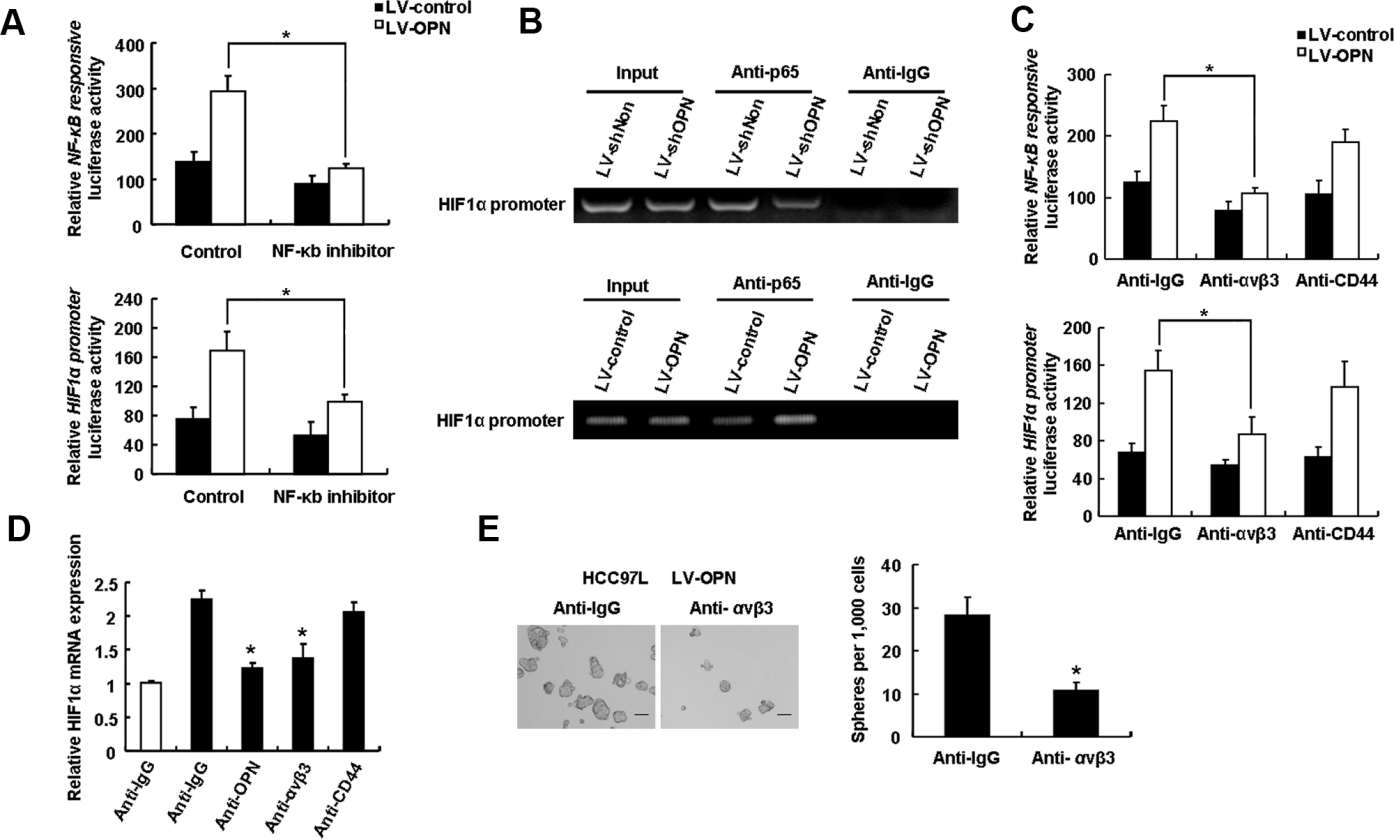
An integrin αvβ3-NF-κB pathway mediates OPN-induced stemness **(A)** NF-kB is activated in response to overexpression of OPN in HCC cells. HCC97L LV-control and LV-OPN cells were transfected with NF-kB-responsive luciferase reporter or HIF-1α promoter luciferase reporter with or without NF-kB inhibitor. Data shown are representative of induction of luciferase activity (*n* = 3; *P < 0.05). **(B)** HCC-LM3 cells were infected with LV-shOPN or LV-OPN or control scrambled lentiviruses. NF-kB binds to the HIF-1α promoter sequence. ChIP was performed using an antibody directed against p65 and a control rabbit IgG antibody. PCR was performed on HIF-1α promoter sequence. **(C)** Blockade of integrin αvβ3 with neutralizing antibodies inhibits OPN-induced activation of NF-kB and HIF-1α transcription factors. **(D)** Blockade of integrin αvβ3 with neutralizing antibodies inhibits OPN-induced transcriptional upregulation of HIF-1α. (E) Treatment of HCC cells that overexpress OPN with neutralizing anti-αvβ3 antibody leads to decreased spheroid formation. Representative images of spheroid formation are shown. *P < 0.05.

In HCC97L cells that overexpressed OPN, NF-kB activity was increased, as was activation of the HIF-1α promoter (Figure 7A). Addition of PDTC, which is an NF-kB inhibitor, resulted in a sharp decrease in NF-kB activity in LV-OPN cells as well as a reduction in activation of the HIF-1α promoter. Together, these results indicate that OPN-induced HIF-1α expression is regulated by NF-kB (Figure 7A). We next sought to determine if NF-kB to binding to the HIF-1α promoter was regulated by OPN. ChIP analysis demonstrated that p65 binding to the HIF-1α promoter was reduced in OPN-deficient cells compared to control cells. Conversely, in cells that overexpress OPN, increased binding of p65 to the HIF-1α promoter was observed (Figure 7B).

We were also interested in determining the factors upstream of this signaling cascade and the receptor to which OPN binds to mediate stemness. We blocked OPN binding with anti-integrin α_v_β_3_ and anti-CD44 antibodies and assessed the effects of blockade on activity of NF-kB and at the HIF-1α promoter. As shown in Figure 7C, blockage of integrin α_v_β_3_ but not of CD44 reversed the effects of OPN overexpression on both activity of NF-kB and activity at the HIF-1α promoter. Consistent with these findings, HIF-1α mRNA levels were decreased in cells treated with antibodies directed against OPN and integrin α_v_β_3_ cells, but not against CD44 (Figure 7D). Moreover, similar to OPN blockade, administration of an anti-α_v_β_3_ antibody inhibited the sphere formation (Figure 7E). These data support a mechanism for OPN regulation that functions through OPN binding to integrin α_v_β_3_, and initiating a signaling cascade that results in upregulation of HIF-1α expression via activation of NF-kB.

## DISCUSSION

HCC is one of the most prevalent and lethal cancers worldwide [32]. Metastasis, tumor recurrence and chemo-resistance are among the leading causes of mortality of patients with HCC. Ten years ago the hypothesis of CSCs being a major contributor to tumor reoccurrence was developed and provided new targets for development of anti-tumor therapies. However, the key molecular and signaling pathways that are involved in maintaining the stemness properties of CSCs are still poorly understood.

Here we demonstrate that the oncoprotein OPN has a critical function in stemness-maintenance of HCC cell lines. We show that expression of OPN protein is upregulated in metastatic HCC cell lines and in primary HCC tumor tissues. Moreover, we report that high OPN expression in HCC tumor tissues is significantly associated with expression of HIF-1α and that OPN binds to integrin α_v_β_3_ and initiates a signaling cascade that results in upregulation of HIF-1α expression via activation of the transcription factor NF-kB, resulting in an increase of tumorigenic capacity of HCC cells.

We propose that OPN is an important oncogene because it is frequently overexpressed in HCC and other types of cancer where it functions to modulate the metastatic potential of various tumors. We have previously shown that OPN inhibits apoptosis of tumor cells via a drug-induced activation of NF-kB thereby facilitating tumorigenesis and metastasis [17]. Importantly, in liver stem/progenitor cell field, OPN has been overlooked as a marker in various rodent models of oval cell induction [33, 34]. Consistent with these findings, in this study we report that OPN is highly expressed in HCC CSCs and functions to promote expression stemness-like factors and tumorigenic properties of HCC. In spheroids, OPN was found coexpressed with hepatocytic and cholangiocytic markers and depletion of OPN resulted in reduced potential to form mixed colonies. Conversely, cells that express high levels of OPN retained the potential for bidirectional differentiation, similar to the pluripotency of stem cells. In our experiments, invasive cells that express high levels of OPN in tumor capsule are positive correlation with patient prognosis feature. Several researches believe OPN originated from inflammatory cells such as macrophage of tumor microenvironment contribute to tumor carcinogenesis and progression [35, 36]. However in our observations, tumor cells which invade in tumor capsule highly expressed OPN (Figure 2A).

According to the traditional definition of CSCs, tumor initiation is a characteristic feature of CSCs [37]. Quiescent CSCs have been proposed to play an important role in tumor growth, recurrence and metastasis. For example, in melanoma, a temporarily distinct subpopulation of slow-cycling cells is required for continuous tumor growth [38]. However, an alternative hypothesis is that proliferating CSCs-like cells (also called stemloids) instead of quiescent stem cells, determine tumor progression, prognosis, therapeutic failures, and resistance to therapy [39, 40]. Here, the observed high expression of OPN in PKH-high and BrdU-label-retaining quiescent cells, as well as in spherical self-renewing cells suggests that OPN maintains the CSCs-like phenotype in quiescent stem cells and proliferating CSCs.

Hypoxia-inducible factors (HIFs) are transcript factors that mediate cellular responses to hypoxia. Several reports have shown that HIFs can promote stem-cell-like phenotypes in normal tissues. Since the emergence of the CSC hypothesis, the function of HIFs in these cells has been investigated extensively. HIF-2α, but not HIF-1α, is induced under hypoxic conditions and upregulation of this factor is critical to maintaining the tumorigenicity of glioma stem cells [41]. Coexpression of the Wnt receptor-Frizzled receptor 6, with HIF1/2α in hypoxic areas of primary human neuroblastomas tumors is characteristic of a population of rare, highly tumorigenic stem-like cells [42]. Interaction of HIF-1α and Notch3, which is required for the expression of the hypoxia-responsive gene Carbonic Anhydrase 9, is a potential target for cancer/stem cell therapy [43]. The HIF1α-Notch pathway is proposed to be essential for maintenance of CSCs in hematological malignancies under conditions of normoxia [44]. It has also been suggested that OPN enhances CSC-like phenotypes and promotes aggressive growth of glioma tumors through activation of HIF-2α, which occurs downstream of OPN binding to CD44 [24]. A recent study also demonstrated hypoxia-induced expression of OPN in breast cancer cells. OPN-induced activation of integrin-linked kinase (ILK)/Akt-mediated NF-kB p65 leads to HIF1α-dependent vascular endothelial growth factor expression and angiogenesis in response to hypoxia [45].

Here our data show that OPN contributes to maintaining HCC stemness via the NF-kB–HIF-1α–BMI1 pathway. OPN expression was significantly associated with levels of HIF-1α in HCC patient samples, which strongly suggests that stemness of HCC cells that is regulated by OPN is intrinsically linked to activation of HIF-1α. BMI1 is an indirect downstream effector of HIF-1α activation and is important to maintenance of stemness-like phenotypes, such as regulation of development of CSCs. There is a large body of evidence to suggest that NF-kB pathway contributes to the development of several types of human cancer including HCC. We and others have previously reported that OPN depletion results in suppression of NF-kB activity. In this work, we have confirmed that OPN regulates NF-kB activity and modulates HIF-1α expression through interactions with integrin αvβ3. These findings are supported by evidence in other cancer types, such as a reported role for NF-kB in CSC expansion in breast cancer [46].

In this article we have demonstrated that OPN maintains CSC-like features via an integrin signaling pathway. CD44 signaling enhances CSC-like phenotypes in glioma [24], however, in our experiments, this pathway does not mediate OPN-HIF-1α-driven self-renewal of HCC cells (Figure 7C, 7D) and further research of the role of CD44 signaling in self-renewal of OPN is warranted. Here, we focused on discerning the function of secreted OPN, however, is it also possible that intracellular OPN contribute toward development and maintenance of CSCs in HCC via mechanisms that are distinct from signaling pathway characterized in the current study.

Our study highlights the role of OPN in maintaining self-renewal and chemo-resistance properties of HCC cells and identifies the OPN–NF-kB–HIF-1α pathway as a potential target for regulating the stemness properties of HCC cells. We also provide evidence that supports using levels of circulating OPN as a novel prognostic marker of HCC possibly in conjunction with other markers of CSCs.

## MATERIALS AND METHODS

### Sphere formation assay and anchorage-independent growth assays

Cells were suspended at a concentration of 1000 cells/mL in serum free medium DMEM/F12 (Invitrogen, Carlsbad, CA, USA) supplemented with 4 μg/mL insulin (Sigma-Aldrich, St Louis, MO, USA), 20 ng/mL epidermal growth factor (PeproTech, Rocky Hill, NJ, USA), 20 ng/mL basic fibroblast growth factor (PeproTech), B27 (1:50; Invitrogen) and 0.4% bovine serum albumin. Cells were plated onto ultralow attachment plates (Corning, Corning, NY, USA). For serial passages of spheres, every 7–12 days spheres were collected, dissociated with trypsin into a single-cell suspension and cultured under conditions as described above. For anchorage-independent growth assays, cells were seeded onto a 24-well plate (500 cells/well) in 0.3% agarose over a 0.6% agarose bottom layer. After three weeks, the numbers of colonies greater than 100 μm in diameter were counted.

### Side population assays

For side population analysis, cells were detached from the dishes with trypsin and suspended in Dulbecco's modified Eagle's medium supplemented with 10% fetal bovine serum at the density of 1×10^6^ cells/mL. The cells were then incubated at 37°C for 90 min with 20 μg/mL Hoechst 33342 (Sigma-Aldrich), either alone or in the presence of 50 μmol/L verapamil (Sigma-Aldrich). Cells were washed, centrifuged and resuspended in ice-cold phosphate-buffered saline (PBS). Then, 1 μg/mL propidium iodide (Sigma-Aldrich) was added and the cells were filtered through a 40 μm cell strainer (BD Biosciences, Franklin Lakes, NJ, USA) to obtain a single-suspension of cells.

### Cell-fate tracing

Cells were labeled with 50 μM PKH26GL (Sigma-Aldrich) according to manufacturer's instructions. For *in vitro* assays, 3×10^4^ labeled HCCLM3 cells were seeded into 24-well plates and immunostaining for OPN was performed after 144 hrs in culture. For *in vivo* assays, 5×10^5^ labeled HCCLM3 cells were subcutaneously injected into nude mice. Six weeks later, the tumors were minced and digested with type IV collagenase (Sigma-Aldrich). Single-cell suspensions were obtained by filtration through a 70 μm filter (BD Biosciences) and immunostaining of OPN was performed. For BrdU-retaining assays, six weeks after intraperitoneal injections with BrdU (10 mg per kg body weight) three times a day for 2 days, the HCCLM3 cells-xenografted nude mice were sacrificed and tumors minced into sections and embedded in paraffin. BrdU-retaining cells were assayed by immunohistochemistry using anti-BrdU antibody (Invitrogen) and goat anti-OPN antibody (R&D Systems). Secondary antibodies were anti-mouse IgG Alexa Fluor 488 and anti-goat IgG Alexa Fluor 555 (Invitrogen).

### Cell viability and chemo-resistance assays

Cells infected with LV-shOPN and LV-shNon were detached, counted and seeded into 96-well plates (5,000 cells/well). After 24 hrs, either 5-FU or PT was added into the medium and incubated for 72 hrs. The media containing either 5-FU or PT was then removed and the cells washed twice with PBS. Cell viability was determined by MTS assay reagent (CellTiter 96 AQueous One Solution Cell Proliferation Assay; Promega, Madison, WI, USA) according to the manufacturer's instructions. The chemo-resistance model was established by intravenously injecting HCCLM3 xenografted-nude mice with PT (once per week for 3 weeks) and resecting the tumors after 3 weeks of treatment. Cells from the resected tumors were then subcutaneously inoculated into PT-treated naïve nude mice, which gave rise to “2° chemo-resistant tumors” after 3 weeks. Generation of 3° and 4° chemo-resistant tumors was achieved through the same serial transfer procedure.

### Mixture colony assay

HCCLM3 and Hep3B cell cultures were dissociated, seeded into 24-well plates (200 cells/well density), and cultured for 10 days. The colonies generated from single cells were analyzed for expression of AFP (hepatic lineage cell marker) and CK19 (cholangiocytic lineage cell marker).

### Chromatin immunoprecipitation (ChIP)

ChIP analysis was performed using the Chromatin Immunoprecipitation Assay Kit (Upstate Biotechnology, Lake Placid, NY, USA). The antibody used for ChIP experiments was anti-p65 (Cell Signaling Technology). PCR was performed with primers for the HIF-1α promoter (forward: 5′-gaacagagagcccagcagag-3′; reverse: 5′-tgtgcactgaggagctgagg-3′) that flanked the NF-kB binding site (−197/188 bp) for 35 cycles with an annealing temperature of at 55°C. We analyzed the amplification products by electrophoresis with 2% agarose gels.

### Animal xenograft tumor

Six-week-old SCID mice were purchased from the Shanghai Experimental Center (CSA, Shanghai, PR China). All mice were housed in a facility with a 12 h light/dark cycle and allowed free access to food and water. Animal care and surgical procedures were approved by the Second Military Medical University Medical Center Institutional Animal Care and Use Committee, as set forth in the ‘Guide for the Care and Use of Laboratory Animals’ published by the National Institutes of Health. For the establishment of xenograft tumors, HCCLM3 cells were suspended at a 1:1 ratio in 200 μL of complete medium and Matrigel (BD Biosciences) and delivered via subcutaneous injections. The size and incidence of subcutaneous tumors were measured and recorded on a weekly basis.

### Clinical specimens

One-hundred-and-twenty-five HCC tumor tissue samples were obtained from the Eastern Hepatobiliary Surgical Hospital (Shanghai, PR China) and Guangxi Cancer Hospital (Nanning, PR China) with informed consent.

### Statistical analysis

The Kaplan-Meier survival analysis was performed to compare patient survival data. The Student's t-test was used to compare data between two groups. The Spearman's rank correlation coefficient and chi-square tests were used to analysis the correlation between the levels of OPN and HIF-1α in HCC patient tumor samples. Differences were considered statistically significant at *P* < 0.05.

### Supporting materials and methods

Detailed methods for cell culture, lentivirus, siRNA assays, immunohistochemistry and immunofluorescence analysis, RNA extraction and reverse transcription PCR, Western blot analysis, Enzyme-linked immunosorbent assays and luciferase-reporter assays are described in Supporting Materials and Methods.

## SUPPLEMENTARY MATERIALS AND METHODS



## Abbreviations

ChIP: chromatin-immunoprecipitation
CSC: cancer stem cell
5-FU: 5-fluorouracil
HCC: hepatocellular carcinoma
HIF-1α: hypoxia-inducible factor 1-alpha
HSC: hematopoietic stem cell
LV: lentivirus
NF-κB: nuclear factor-kappa B
OPN: osteopontin
PBS: phosphate-buffered saline
PE: phycoerythrin
PT: cisplatin
shRNA: small hairpin RNA
siRNA: small interfering RNA

## References

[1] Siegel R, Naishadham D, Jemal A (2012). Cancer statistics. CA Cancer J Clin.

[2] Kassahun WT, Fangmann J, Harms J, Hauss J, Bartels M (2006). Liver resection and transplantation in the management of hepatocellular carcinoma: a review. Exp Clin Transplant.

[3] Aguayo A, Patt YZ (2001). Nonsurgical treatment of hepatocellular carcinoma. Semin Oncol.

[4] Llovet JM, Bruix J (2003). Systematic review of randomized trials for unresectable hepatocellular carcinoma: Chemoembolization improves survival. Hepatology.

[5] Visvader JE, Lindeman GJ (2008). Cancer stem cells in solid tumours: accumulating evidence and unresolved questions. Nat Rev Cancer.

[6] Arai F, Hirao A, Ohmura M, Sato H, Matsuoka S, Takubo K, Ito K, Koh GY, Suda T (2004). Tie2/angiopoietin-1 signaling regulates hematopoietic stem cell quiescence in the bone marrow niche. Cell.

[7] Meng S, Tripathy D, Frenkel EP, Shete S, Naftalis EZ, Huth JF, Beitsch PD, Leitch M, Hoover S, Euhus D, Haley B, Morrison L, Fleming TP (2004). Circulating tumor cells in patients with breast cancer dormancy. Clin Cancer Res.

[8] Haraguchi N, Ishii H, Mimori K, Tanaka F, Ohkuma M, Kim HM, Akita H, Takiuchi D, Hatano H, Nagano H, Barnard GF, Doki Y, Mori M (2010). CD13 is a therapeutic target in human liver cancer stem cells. J Clin Invest.

[9] Ma S, Lee TK, Zheng BJ, Chan KW, Guan XY (2008). CD133+ HCC cancer stem cells confer chemoresistance by preferential expression of the Akt/PKB survival pathway. Oncogene.

[10] Ma S, Tang KH, Chan YP, Lee TK, Kwan PS, Castilho A, Ng I, Man K, Wong N, To KF, Zheng BJ, Lai PB, Lo CM (2010). miR-130b Promotes CD133(+) liver tumor-initiating cell growth and self-renewal via tumor protein 53-induced nuclear protein 1. Cell Stem Cell.

[11] Yang ZF, Ho DW, Ng MN, Lau CK, Yu WC, Ngai P, Chu PW, Lam CT, Poon RT, Fan ST (2008). Significance of CD90+ cancer stem cells in human liver cancer. Cancer Cell.

[12] Yamashita T, Ji J, Budhu A, Forgues M, Yang W, Wang HY, Jia H, Ye Q, Qin LX, Wauthier E, Reid LM, Minato H, Honda M (2009). EpCAM-positive hepatocellular carcinoma cells are tumor-initiating cells with stem/progenitor cell features. Gastroenterology.

[13] Lee TK, Castilho A, Cheung VC, Tang KH, Ma S, Ng IO (2011). CD24(+) liver tumor-initiating cells drive self-renewal and tumor initiation through STAT3-mediated NANOG regulation. Cell Stem Cell.

[14] Chiba T, Kita K, Zheng YW, Yokosuka O, Saisho H, Iwama A, Nakauchi H, Taniguchi H (2006). Side population purified from hepatocellular carcinoma cells harbors cancer stem cell-like properties. Hepatology.

[15] Rittling SR, Chambers AF (2004). Role of osteopontin in tumour progression. Br J Cancer.

[16] Rangaswami H, Bulbule A, Kundu GC (2006). Osteopontin: role in cell signaling and cancer progression. Trends Cell Biol.

[17] Zhao J, Dong L, Liu B, Wu GB, Xu DM, Chen JJ, Li K, Tong X, Dai JX, Yao S, Wu MC, Guo YJ (2008). Down-regulation of osteopontin suppresses growth and metastasis of hepatocellular carcinoma via induction of apoptosis. Gastroenterology.

[18] Wai PY, Mi Z, Gao C, Guo H, Marroquin C, Kuo PC (2006). Ets-1 and runx2 regulate transcription of a metastatic gene, osteopontin, in murine colorectal cancer cells. J Biol Chem.

[19] Das R, Mahabeleshwar GH, Kundu GC (2003). Osteopontin stimulates cell motility and nuclear factor kappaB-mediated secretion of urokinase type plasminogen activator through phosphatidylinositol 3-kinase/Akt signaling pathways in breast cancer cells. J Biol Chem.

[20] Rangaswami H, Bulbule A, Kundu GC (2004). Nuclear factor-inducing kinase plays a crucial role in osteopontin-induced MAPK/IkappaBalpha kinase-dependent nuclear factor kappaB-mediated promatrix metalloproteinase-9 activation. J Biol Chem.

[21] Rangaswami H, Bulbule A, Kundu GC (2005). JNK1 differentially regulates osteopontin-induced nuclear factor-inducing kinase/MEKK1-dependent activating protein-1-mediated promatrix metalloproteinase-9 activation. J Biol Chem.

[22] Stier S, Ko Y, Forkert R, Lutz C, Neuhaus T, Grunewald E, Cheng T, Dombkowski D, Calvi LM, Rittling SR, Scadden DT (2005). Osteopontin is a hematopoietic stem cell niche component that negatively regulates stem cell pool size. J Exp Med.

[23] Nilsson SK, Johnston HM, Whitty GA, Williams B, Webb RJ, Denhardt DT, Bertoncello I, Bendall LJ, Simmons PJ, Haylock DN (2005). Osteopontin, a key component of the hematopoietic stem cell niche and regulator of primitive hematopoietic progenitor cells. Blood.

[24] Pietras A, Katz AM, Ekstrom EJ, Wee B, Halliday JJ, Pitter KL, Werbeck JL, Amankulor NM, Huse JT, Holland EC (2014). Osteopontin-CD44 signaling in the glioma perivascular niche enhances cancer stem cell phenotypes and promotes aggressive tumor growth. Cell Stem Cell.

[25] Haraguchi N, Utsunomiya T, Inoue H, Tanaka F, Mimori K, Barnard GF, Mori M (2006). Characterization of a side population of cancer cells from human gastrointestinal system. Stem Cells.

[26] Golebiewska A, Brons NH, Bjerkvig R, Niclou SP (2011). Critical appraisal of the side population assay in stem cell and cancer stem cell research. Cell Stem Cell.

[27] Shi GM, Xu Y, Fan J, Zhou J, Yang XR, Qiu SJ, Liao Y, Wu WZ, Ji Y, Ke AW, Ding ZB, He YZ, Wu B (2008). Identification of side population cells in human hepatocellular carcinoma cell lines with stepwise metastatic potentials. J Cancer Res Clin Oncol.

[28] Dontu G, Abdallah WM, Foley JM, Jackson KW, Clarke MF, Kawamura MJ, Wicha MS (2003). In vitro propagation and transcriptional profiling of human mammary stem/progenitor cells. Genes Dev.

[29] Kim J, Ki SS, Lee SD, Han CJ, Kim YC, Park SH, Cho SY, Hong YJ, Park HY, Lee M, Jung HH, Lee KH, Jeong SH (2006). Elevated plasma osteopontin levels in patients with hepatocellular carcinoma. Am J Gastroenterol.

[30] Yang MH, Hsu DS, Wang HW, Wang HJ, Lan HY, Yang WH, Huang CH, Kao SY, Tzeng CH, Tai SK, Chang SY, Lee OK, Wu KJ (2010). Bmi1 is essential in Twist1-induced epithelial-mesenchymal transition. Nat Cell Biol.

[31] Bonello S, Zahringer C, BelAiba RS, Djordjevic T, Hess J, Michiels C, Kietzmann T, Gorlach A (2007). Reactive oxygen species activate the HIF-1alpha promoter via a functional NFkappaB site. Arterioscler Thromb Vasc Biol.

[32] Siegel R, Ma J, Zou Z, Jemal A (2014). Cancer statistics. CA Cancer J Clin.

[33] Syn WK, Choi SS, Liaskou E, Karaca GF, Agboola KM, Oo YH, Mi Z, Pereira TA, Zdanowicz M, Malladi P, Chen Y, Moylan C, Jung Y (2011). Osteopontin is induced by hedgehog pathway activation and promotes fibrosis progression in nonalcoholic steatohepatitis. Hepatology.

[34] Wang X, Lopategi A, Ge X, Lu Y, Kitamura N, Urtasun R, Leung TM, Fiel MI, Nieto N (2014). Osteopontin induces ductular reaction contributing to liver fibrosis. Gut.

[35] Rao G, Wang H, Li B, Huang L, Xue D, Wang X, Jin H, Wang J, Zhu Y, Lu Y, Du L, Chen Q (2013). Reciprocal interactions between tumor-associated macrophages and CD44-positive cancer cells via osteopontin/CD44 promote tumorigenicity in colorectal cancer. Clin Cancer Res.

[36] Kale S, Raja R, Thorat D, Soundararajan G, Patil TV, Kundu GC (2014). Osteopontin signaling upregulates cyclooxygenase-2 expression in tumor-associated macrophages leading to enhanced angiogenesis and melanoma growth via alpha9beta1 integrin. Oncogene.

[37] Clarke MF, Dick JE, Dirks PB, Eaves CJ, Jamieson CH, Jones DL, Visvader J, Weissman IL, Wahl GM (2006). Cancer stem cells—perspectives on current status and future directions: AACR Workshop on cancer stem cells. Cancer Res.

[38] Roesch A, Fukunaga-Kalabis M, Schmidt EC, Zabierowski SE, Brafford PA, Vultur A, Basu D, Gimotty P, Vogt T, Herlyn M (2010). A temporarily distinct subpopulation of slow-cycling melanoma cells is required for continuous tumor growth. Cell.

[39] Blagosklonny MV (2006). Target for cancer therapy: proliferating cells or stem cells. Leukemia.

[40] Blagosklonny MV (2007). Cancer stem cell and cancer stemloids: from biology to therapy. Cancer Biol Ther.

[41] Li Z, Bao S, Wu Q, Wang H, Eyler C, Sathornsumetee S, Shi Q, Cao Y, Lathia J, McLendon RE, Hjelmeland AB, Rich JN (2009). Hypoxia-inducible factors regulate tumorigenic capacity of glioma stem cells. Cancer Cell.

[42] Cantilena S, Pastorino F, Pezzolo A, Chayka O, Pistoia V, Ponzoni M, Sala A (2011). Frizzled receptor 6 marks rare, highly tumourigenic stem-like cells in mouse and human neuroblastomas. Oncotarget.

[43] Shareef MM, Udayakumar TS, Sinha VK, Saleem SM, Griggs WW (2013). Interaction of HIF-1alpha and Notch3 Is Required for the Expression of Carbonic Anhydrase 9 in Breast Carcinoma Cells. Genes Cancer.

[44] Wang Y, Liu Y, Malek SN, Zheng P, Liu Y (2011). Targeting HIF1alpha eliminates cancer stem cells in hematological malignancies. Cell Stem Cell.

[45] Raja R, Kale S, Thorat D, Soundararajan G, Lohite K, Mane A, Karnik S, Kundu GC (2014). Hypoxia-driven osteopontin contributes to breast tumor growth through modulation of HIF1alpha-mediated VEGF-dependent angiogenesis. Oncogene.

[46] Kendellen MF, Bradford JW, Lawrence CL, Clark KS, Baldwin AS (2014). Canonical and non-canonical NF-kappaB signaling promotes breast cancer tumor-initiating cells. Oncogene.

