# Supplementary material for: Osteopontin promotes a cancer stem cell-like phenotype in hepatocellular carcinoma cells via an integrin–NF-κB–HIF-1α pathway
Source: Oncotarget. 2015 Feb 25;6(9):6627–40. doi: 10.18632/oncotarget.3113 (PMC4466639; doi:10.18632/oncotarget.3113)
Supplement: Supplementary file 1 [file oncotarget-06-6627-s001.pdf]

SUPPLEMENTARY MATERIALS AND METHODS

Cell culture, lentivirus (LV), small interfering (si) RNA assays

PLC/PRF/5 and Hep3B cell lines were purchased from American Type Culture Collection (ATCC, Manassas, VA, USA). HCC97L, HCC97H and HCCLM3 cell lines were obtained from the Liver Cancer Institute of Zhong Shan Hospital, Fudan University (Shanghai, PR China). Huh-7 and HL7702 cell lines were obtained from the Cell Bank of Shanghai Institutes for Biological Sciences, Chinese Academy of Sciences (Shanghai, PR China). Cells were grown in Dulbecco's modified Eagle's medium (Gibco Life Technologies, Carlsbad, CA, USA) supplemented with 10% (vol/vol) fetal bovine serum (Hyclone, Logan City, UT, USA) in a humidified incubator at 37°C in 5% CO_2 atmosphere. For OPN-depleted-conditioned media, complete media from cultures of HCCLM3 spheres was collected and incubated overnight with anti-OPN antibody (R&D Systems, Minneapolis, MN, USA). The mixture was applied to a protein A/G agarose matrix for 2 hrs and centrifuged for 1 min at 3000 rpm, and the resultant supernatant collected. We generated LV vectors encoding *OPN* and the empty control, small hairpin (sh)RNAs targeting *OPN* and scramble shRNA, and used them as previously described [1]. The LV vectors encoding HIF-1α, BMI1 were purchased from Fulengen (Guangzhou, PR China). siRNAs against HIF-1α, BMI1 and scrambled siNC were purchased from Genepharma (Shanghai, PR China) and all siRNAs sequences are provided in the Supplementary Table S2.

Immunohistochemistry and immunofluorescence analysis

For paraffin-embedded tissues, 4 μm-thick paraffin-embedded sections were deparaffinized in xylene and rehydrated in an ethanol (descending) gradient. The sections were then processed for antigen retrieval by heating in 0.01 M (pH 6.0) citric acid buffer. The samples were then incubated with goat anti-OPN antibody (5 μg/mL; R&D Systems) or mouse anti-HIF-1α antibody (1:100; Neomarkers, Fremont, CA, USA). Anti-goat or anti-mouse peroxidase-conjugated secondary antibodies (Santa Cruz Biotechnology, Santa Cruz, CA, USA) were applied. Color development was performed with diaminobenzidine peroxidase substrate. Finally, slides were counterstained with hematoxylin (Sigma-Aldrich). For cells and spheres immunofluorescence analysis, collected cells or spheres were collected and fixed with 4% paraformaldehyde for 15 min. After blockade with 0.5% triton x-100, the samples were incubated with primary antibody at 4°C overnight. We employed the following antibodies: rabbit anti-human A-fetoprotein (1:50; Dako, Glostrup, Denmark), mouse anti-human Cytokine 19 (1:50; Dako), rabbit anti-human Oct4 (1:100; Abcam, Cambridge, MA, USA). For secondary antibody, anti-mouse IgG Alexa Fluor 488, anti-goat IgG Alexa Fluor 555, anti-rabbit IgG Alexa Fluor 555 and anti-goat IgG Alexa Fluor 488 (Invitrogen) were used. Finally, the cells were counterstained with DAPI (Dojindo Laboratories, Kumamoto, Japan) for 5 min at room temperature and observed by fluorescent microscope (Olympus, Tokyo, Japan).

RNA extraction and reverse transcription PCR

Total RNA was extracted from cells or tissues using the Trizol Reagent (Invitrogen) and used (1 μg from each sample), along with random primers, to synthesize cDNA with reagents and instructions from the First-Strand cDNA Synthesis Kit (Takara, Shiga, Japan). The samples were denatured at 94°C for 5 min, then subjected to 22–38 cycles of denaturation at 94°C for 30 sec, annealing at 52–58°C for 30 sec, and extension at 72°C for 30 sec, with the final extension at 72°C for 5 min. Real time PCR was performed using the 7500 Real Time System (Applied Biosystems Inc.). β-actin was amplified for use as an endogenous control. The primer sequences used in this study are listed in the Supplementary Table S3.

Western blot analysis

Total cell lysates were prepared as previously described [2]. Equal amounts of proteins were loaded and separated by sodium dodecyl sulfate polyacrylamide gel electrophoresis and transferred onto polyvinylidene difluoride membranes. The primary antibodies used in western blotting were: anti-HIF-1α, (Cell Signaling Technology, Danvers, MA, USA), anti-OPN, anti-BMI1 (R&D Systems), and GAPDH (endogenous control; KangChen Bio-tech, Shanghai, PR China). Horseradish peroxidase-conjugated anti-mouse or rabbit or goat secondary antibodies were purchased from Santa Cruz Biotechnology. After probing with primary and secondary antibody, antigen–antibody complexes were visualized using the enhanced chemiluminescence reagent, Supersignal (Thermo Fisher Scientific Inc., Rockford, IL, USA).

Enzyme-linked immunosorbent assays

2×10^5 cells were seeded into a 6-well plate and viable cell numbers were assessed every 12 hrs by trypan-blue staining. Concentrations of OPN in conditioned media were measured and calculated as previously described [2] with a quantitative immunoassay enzyme-linked immunosorbent assay kit (R&D Systems). For each assay, triplicate samples were analyzed.

Luciferase reporter assays

Cells were transfected with an NF-κB-driven luciferase plasmid or a HIF-1α-538 promoter luciferase plasmid. pRL-TK was cotransfected as a control for transfection efficiency. The NF-κB inhibitor PDTC (Beyotime, Haimen, Jiangsu, China) or anti-$\alpha_v\beta_3$ antibody (eBioscience) was added to the cell culture medium simultaneously with the transfection of plasmids. Twenty-four hrs after transfection, the cells were collected and luciferase activities were measured using the Dual Luciferase Reporter Assay System (Promega).

REFERENCES

1. Zhao J, Lu B, Xu H, Tong X, Wu G, Zhang X, Liang A, Cong W, Dai J, Wang H, Wu M, Guo Y. Thirty-kilodalton Tat-interacting protein suppresses tumor metastasis by inhibition of osteopontin transcription in human hepatocellular carcinoma. Hepatology. 2008; 48:265–275.
2. Zhao J, Dong L, Liu B, Wu GB, Xu DM, Chen JJ, Li K, Tong X, Dai JX, Yao S, Wu MC, Guo YJ. Down-regulation of osteopontin suppresses growth and metastasis of hepatocellular carcinoma via induction of apoptosis. Gastroenterology. 2008; 135:956–968.

SUPPLEMENTARY FIGURES AND TABLES

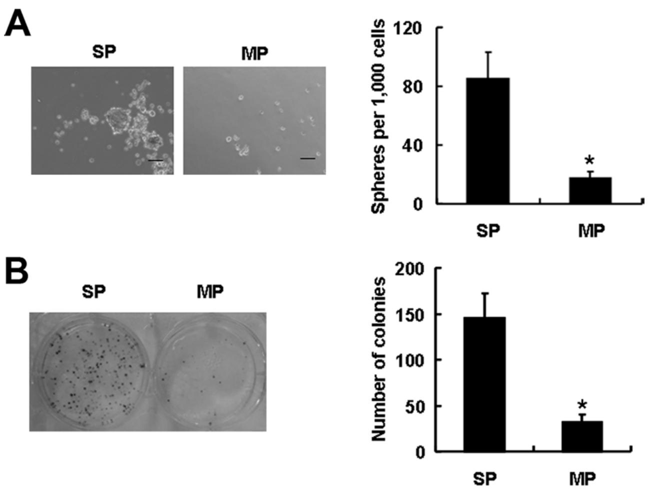

Supplementary Figure S1: (A) Side population fraction cells generated increased numbers of spheres or (B) colonies compared with cells in the main population fraction. Representative results from at least two independent experiments are shown.

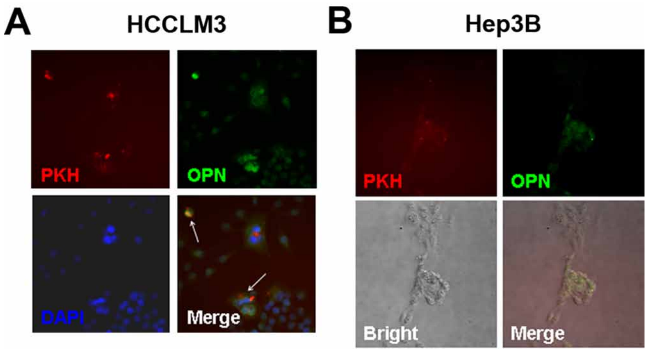

Supplementary Figure S2: OPN staining of PKH labeled HCCLM3 (A) and Hep3B (B) cells after 144 hrs of culture.

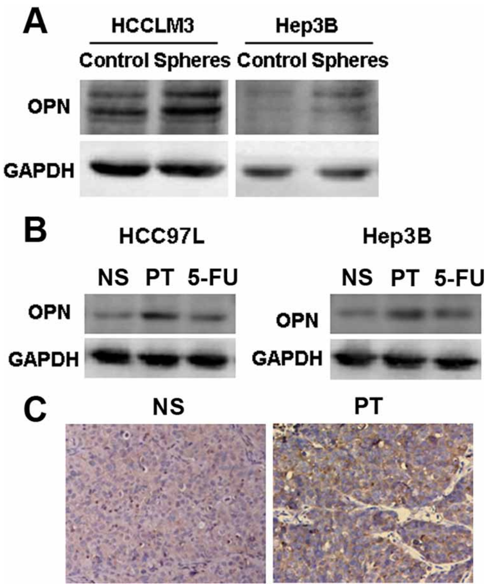

Supplementary Figure S3: (A) OPN protein levels are higher in spheroid cells than in adherent cells in both HCCLM3 and Hep3B cell lines. **(B)** OPN expression is higher in 5-FU and PT treated cells versus control cells. **(C)** Immunohistochemistry demonstrates that OPN has higher expression in PT treated-tumor than in saline-treated tumors. Original magnification ×20.

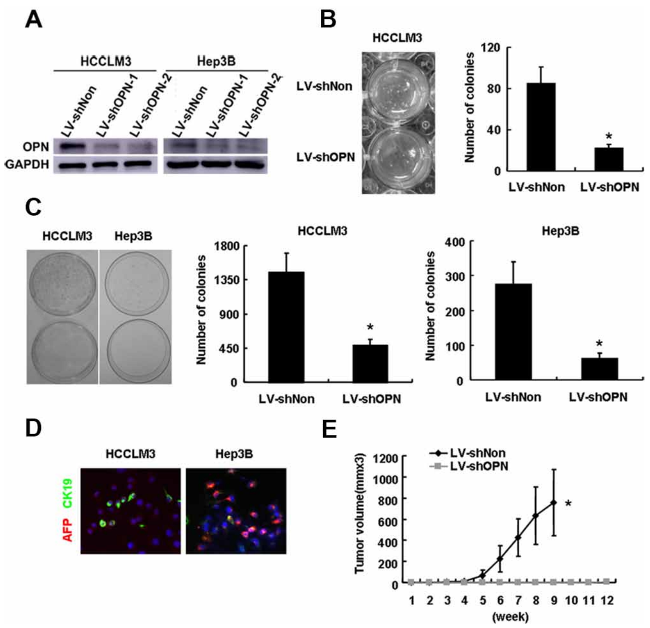

Supplementary Figure S4: (A) The knockdown of OPN was confirmed by western blot analysis in HCCLM3 and Hep3B cell lines. Two target sites were selected for knockdown of OPN by shRNA. Target site 2 was more effective and was chosen for further study. **(B)** Cells knocked down for OPN had a lower potential to form 3D-colonies in soft agar or **(C)** colonies which derive from singe cells than control cells. **(D)** Representative mixed colonies of HCCLM3 and Hep3B cells. **(E)** HCCLM3 cells in which OPN expression was knocked out were unable to form tumors *in vivo* compared to HCCLM3 cells with endogenous expression levels of OPN. Tumor volume was monitored and measured bi-weekly. $^*P < 0.05$.

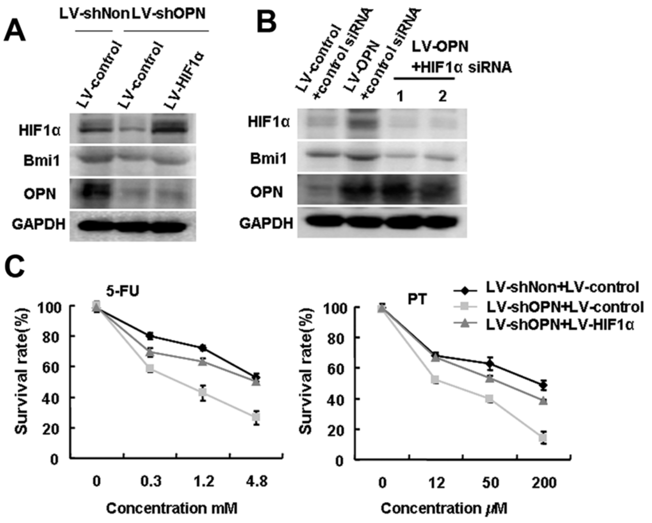

Supplementary Figure S5: (A) Expression of HIF-1α, BMI1 and OPN proteins in HCCLM3 cells infected by lentivirus and transfected with LV-HIF1α or LV-control were analyzed by western blot assay. (B) Expression of HIF-1α, BMI1 and OPN proteins in HCC97L cells infected by lentivirus and transfected with HIF-1α siRNA or scrambled siRNA (control siRNA) were analyzed by western blot assay. **(C)** Increasing concentrations of 5-FU and PT were added in HCCLM3 LV-shNon or LV-shOPN cells infected by LV-HIF-1α or control lentivirus. After 48 hr the cell survival rates were determined by MTS assay. $P < 0.01$.

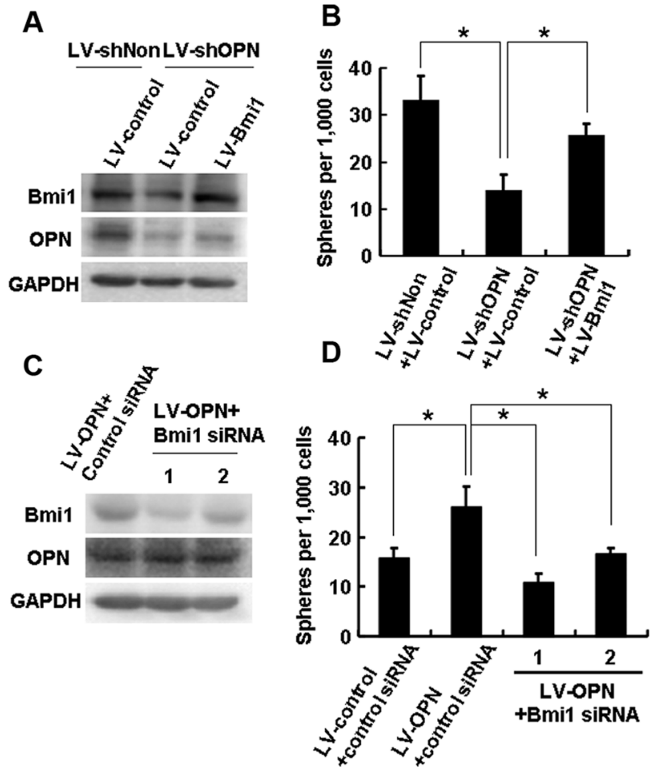

Supplementary Figure S6: (A) Expression of BMI1 and OPN in HCCLM3 cells infected by lentivirus and transfected with LV-*BMI1* or LV-control were analyzed by western blot assay. (B) Quantification of the spheroids from HCCLM3 infected by lentivirus indicated. **(C)** Expression of BMI1 and OPN in HCC97L cells infected by lentivirus and transfected with BMI1 siRNA or scrambled siRNA (control siRNA) were analyzed by western blot assay. **(D)** Quantification of the spheroids from HCC97L with lentivirus and siRNA indicated. The data are reported as mean ± SD. $^*P < 0.05$.

Supplementary Table S1: Summary of clinicopathologic variables

Feature	Number of patients
Patients	125
Gender	
Male	111
Female	14
Age(years)	18–75, median = 46
HbsAg	
Positive	118
Negative	7
AFP(ng/ml)	
≤400	57
>400	68
Cirrhosis	
–	23
+	102
Tumor volume(cm)	1.4–24, median = 6.5
AJCC stage	
I–II	73
III–IV	52
Metastasis	
–	68
+	57
Time of follow-up(months)	1–60, median = 32

Supplementary Table S2: Sequences of siRNAs used in this study

	Sequence	
siRNA	Sense, 5′–3′	Antisense, 5′–3′
siNC	UUCUCCGAACGUGUCACGUTT	ACGUGACACGUUCGGAGAATT
siHIF1α-1	GCCGCUCAAUUUAUGAAUATT	UAUUCAUAAAUUGAGCGGCTT
siHIF1α-2	GCCUCUUUGACAAACUUAATT	UUAAGUUUGUCAAAGAGGCTT
siBMI1-1	CCAGACCACUACUGAAUAUTT	AUAUUCAGUAGUGGUCUGGTT
siBMI1-2	GGAUCGGAAAGUAAACAAATT	UUUGUUUACUUUCCGAUCCTT

Supplementary Table S3: Primers for PCR used in this study

Primers		Sequence, 5′→3′
β-actin	F	CGTGGACATCCGTAAAGACC
	R	ACATCTGCTGGAAGGTGGAC
OPN	F	CTCCATTGACTCGAACGACTC
	R	CAGGTCTGCGAAACTTCTTAGAT
CK19	F	AACGGCGAGCTAGAGGTGA
	R	GGATGGTCGTGTAGTAGTGGC
Nanog	F	TTTGTGGGCCTGAAGAAAACT
	R	AGGGCTGTCCTGAATAAGCAG
Sox2	F	GCCGAGTGGAAACTTTTGTCG
	R	GGCAGCGTGTACTTATCCTTCT
HIF1α	F	GGCGCGAACGACAAGAAAAAG
	R	CCTTATCAAGATGCGAACTCACA
BMI1	F	CGTGTATTGTTCGTTACCTGGA
	R	TTCAGTAGTGGTCTGGTCTTGT
Oct4	F	CTGGGTTGATCCTCGGACCT
	R	CCATCGGAGTTGCTCTCCA
ABCG2	F	CAGGTGGAGGCAAATCTTCGT
	R	ACCCTGTTAATCCGTTCGTTTT
Notch-1	F	GAGGCGTGGCAGACTATGC
	R	CTTGTACTCCGTCAGCGTGA
ABCB1	F	TTGCTGCTTACATTCAGGTTTCA
	R	AGCCTATCTCCTGTCGCATTA
ABCC1	F	CTCTATCTCTCCCGACATGACC
	R	AGCAGACGATCCACAGCAAAA
